# Outbreak of Enterovirus D68 in Young Children, Brescia, Italy, August to November 2024

**DOI:** 10.1002/jmv.70372

**Published:** 2025-04-29

**Authors:** Serena Messali, Anna Bertelli, Laura Dotta, Marta Giovanetti, Leonardo Sclavi, Giulia Venneri, Massimo Ciccozzi, Raffaele Badolato, Arnaldo Caruso, Francesca Caccuri

**Affiliations:** ^1^ Section of Microbiology, Department of Molecular and Translational Medicine University of Brescia Brescia Italy; ^2^ Institute of Microbiology ASST‐Spedali Civili, Brescia Brescia Italy; ^3^ Department of Clinical and Experimental Sciences, Pediatric Clinic and Institute for Molecular Medicine “A. Nocivelli” University of Brescia and ASST‐Spedali Civili di Brescia Brescia Italy; ^4^ Sciences and Technologies for Sustainable Development and One Health University of Campus Bio‐Medico Rome Italy; ^5^ Instituto René Rachou Fundação Oswaldo Cruz Belo Horizonte Minas Gerais Brazil; ^6^ Unit of Medical Statistics and Molecular Epidemiology University of Campus Bio‐Medico Rome Italy

**Keywords:** children, clinical presentation, enterovirus D68, epidemiology, phylogenetic analysis

## Abstract

Enterovirus D68 (EV‐D68) is responsible for a plethora of clinical manifestations ranging from asymptomatic infections to severe respiratory symptoms and neurological disorders. EV‐D68 was first detected in children with pneumonia in 1962 and, from then, only sporadic cases were reported until 2014, when outbreaks were notified across the world. After the withdrawal of preventive measures against SARS‐CoV‐2, a significant increase in EV‐D68 infections has been reported in 2021–2022. A surveillance program to evaluate the incidence of enterovirus/rhinovirus (EV/RV) infections was implemented at the Brescia Civic Hospital, Italy. Fifty‐five EV/RV‐positive respiratory samples, belonging to pediatric patients, were subjected to NGS. We observed that 61.8% of samples were positive for EV, with EV‐D68 as the most prevalent genotype predominantly detected between August and November 2024. Phylogenetic analysis revealed that EV‐D68 sequences formed two monophyletic clades corresponding to the A2 and B3 lineages, highlighting their recent introduction in Italy. Interestingly, 40% of pediatric EV‐D68 infections were detected with at least one other EV/RV. Our study highlights the crucial role played by genomic surveillance of respiratory infections to monitor the circulation of emerging and re‐emerging viruses, as well as their evolution. This will be fundamental to enable prompt intervention strategies.

## 1 Introduction

Enterovirus D68 (EV‐D68) is a non‐enveloped, positive‐sense, single‐stranded RNA virus belonging to the Enterovirus genus of the *Picornaviridae* family [1, 2]. Four distinct clades of EV‐D68 (A–D) have been described in addition to subclades A1, A2, B1, B2, and B3 [3]. EV‐D68 was first detected in 1962 in children with pneumonia [4]. After that, only sporadic EV‐D68 positive cases have been reported until 2014 and 2016, when two large outbreaks of respiratory disease spread across the USA [1] and several European countries, sustained by strains belonging to multiple EV‐D68 clades [5, 6]. In particular, the 2016 outbreak was superimposed by an unprecedented cluster of acute flaccid myelitis (AFM) cases, whose symptoms were clinically similar to poliomyelitis [6]. Once the association between EV‐D68 infection and AFM was confirmed [7, 8, 9], this caused public health concern and provided EV‐D68 surveillance in several countries. In the same period, circulation of EV‐D68 strains genetically related to the North American ones was documented, although at lower levels, also in Europe [10]. Subsequently, in temperate regions, EV‐D68 outbreaks took place in late summer‐autumn of 2016 and 2018, following a biennial pattern [1]. This trend was interrupted by the upsurge of EV‐D68 cases reported in Europe in 2019 [3] and by the onset of the SARS‐CoV‐2 pandemic in 2020 [11]. After the withdrawal of preventive measures to counteract SARS‐CoV‐2 spread, which has contributed to the selection of an EV immune‐naive population, a significant increase in EV‐D68 infections was reported during winter season 2021–2022 [11].

In 2008, EV‐D68 was first detected in Italy, in patients with respiratory syndromes [12, 13]. Despite two AFM cases associated with EV‐D68 infection were recognized between 2016 and 2018 in Italy [14, 15, 16], more recent outbreaks that occurred in Lombardy, northern Italy, did not give rise to cases of acute flaccid paralysis (AFP) or AFM [17, 18]. Interestingly, the new upsurge of EV‐D68 occurred in Lombardy from January to September 2024, was found to affect primarily adults > 15 years rather than young children [18]. Following this finding, the authors hypothesized a change in the epidemiological characteristics of EV‐D68, possibly linked to viral genome mutations and evolution, a different level of population immunity or some other unforeseen characteristics [18].

To better evaluate whether the involvement of pediatric patients occurred during the EV‐D68 outbreak in Lombardy, we retrospectively confirmed, by next generation sequencing (NGS), EV‐positive samples from patients admitted at the Emergency Room (ER) or hospitalized at the pediatric clinic of a major tertiary center and research hospital in Brescia, from May to December 2024. Here, according to previous studies [3, 19, 20, 21], we highlight the outbreak of EV‐D68 infections in young children, and describe both molecular characteristics of the virus and clinical presentation of the confirmed EV‐D68 cases of infection.

## 2 Materials and Methods

### 2.1 Specimens Types and Collection

The study was carried out on data collected at the Brescia Civic Hospital (Brescia, Lombardy, Italy) from May to December 2024. Respiratory samples, such as nasopharyngeal swabs (NPS), nasopharyngeal aspirates (NPA), and one cerebrospinal fluid (CSF) were obtained from children admitted at the ER or hospitalized at the Brescia Civic Hospital. During molecular routine evaluations, 55 samples tested positive for EV/RV with cycle threshold (Ct) value under 30. Total nucleic acid was extracted from each sample using the ELITe InGenius total nucleic acid isolation protocol (ELITech), following the manufacturer's instructions. Amplification was performed on the ELITe InGenius PCR platform (ELITech). The ampliCube Respiratory Viral Panel 4 (Mikrogen Diagnostik) or the FTD Respiratory pathogens 21 (Fast Track Diagnostic) was chosen for the evaluation of EV/RV positivity. Following their routine evaluations, specimens were frozen at −80°C until the day of processing.

### 2.2 Viral RNA Extraction, Reverse Transcription, and Amplification of EV/RV‐Positive Samples

Viral RNA was automatically extracted from 200 µL of sample on ELITe InGenius (ELITech) with magnetic beads, eluated in 100 μL and stored at −80°C until use. Then, the VP2‐VP4 genes were retro‐transcribed and amplified with the SuperScript IV One‐Step RT‐PCR System (ThermoFisher Scientific) in a 50‐µL reaction, specifically containing 12 µL of viral RNA, 25 µL of 2X Platinum SuperFi RT‐PCR Master Mix, 0.5 µL of SuperScript IV RT Mix, 0.75 µM of OS458 (sense primer: 5′‐CCGGCCCCTGAATGYGGCTAA‐3′), 0.9 µM of OAS1125 (antisense primer: 5′‐ACATRTTYTSNCCAAANAYDCCCAT‐3′) [22]. The One‐Step RT‐PCR conditions were as follows: 55°C for 10′ for reverse transcription, 98°C for 2 min for the RT inactivation/initial denaturation step, followed by 40 cycles (98°C for 10 s, 58°C for 10 s, 72°C for 30 s) and a final cycle at 72°C for 5 min. Subsequently, a Nested PCR was performed with the SuperScript IV One‐Step RT‐PCR System (ThermoFisher Scientific) in a 50 µL reaction, specifically containing 12 µL of the One‐Step RT‐PCR product previously obtained, 25 µL of 2X Platinum SuperFi RT‐PCR Master Mix, 0.5 µL of SuperScript IV RT Mix, 0.75 µM of IS547 (sense primer: 5′‐ACCRACTACTTTGGGTGTCCGTG‐3′), 0.9 µM of IAS1087 (antisense primer: 5′‐TCWGGHARYTTCCAMCACCANCC‐3′). The Nested PCR amplification conditions were the same employed for the One‐Step RT‐PCR. Afterwards, nested‐PCR products were checked on 1.0% agarose gel, were purified through Wizard SV Gel and PCR Clean‐Up System (Promega) according to the manufacturer's instructions, and quantified using the Qubit DNA HS Assay Kit (ThermoFisher Scientific).

### 2.3 NGS and Genotyping

NGS was performed with an amplicon‐target approach. In brief, 15 μL of the amplified Nested‐PCR was used in the tagmentation reaction using Nextera chemistry (Illumina) to yield fragments > 150 bp, according to the protocol. The tagmented library underwent 8 cycles of PCR with indexed primers (IDT Technologies), followed by purification using AMPure XP beads. Purified library was quantified with the Qubit Fluorometer (ThermoFisher Scientific) and loaded in a V2 300‐cycle sequencing cartridge, to perform sequencing on the MiSeq platform (Illumina). Raw data were checked for quality using FastQC (https://www.bioinformatics.babraham.ac.uk/projects/fastqc/), were trimmed with Trimmomatic version 0.39 [23], and then, the derived sequences were analysed with Geneious software (v.11.1.5) (Biomatters Ltd.). Genotype assignment was performed with the online tool Genome Detective [24], with default parameters. Then, the resulting draft assemblies were subjected to BLASTn, Enterovirus Genotyping tool (https://www.rivm.nl/mpf/typingtool/enterovirus/) and MEGABLAST algorithms for nucleotide similarity searches in the National Center for Biotechnology Information (NCBI). EV and RV nomenclature was assigned according to Simmonds and colleagues 2020 [25].

### 2.4 Phylogenetic Analysis

A total of 25 novel isolates obtained from this study were analyzed alongside 1286 high‐quality EV‐D68 genomes retrieved from GenBank (NCBI), which included information on location and date of isolation. The reference genome set encompasses samples from Africa, Asia, Europe, Italy, North America and Oceania, spanning subclades A1, A2, B, B1, B2, B3, and C. These genomes cover a timeframe from November 2002 to January 2024. Additionally, the dataset includes the most recent strains isolated in Italy (*n* = 27) in 2024 [18]. Sequence alignment was performed using MAFFT and edited with AliView [26, 27]. The phylogenetic tree was constructed using IQ‐TREE 2 under the GTR + G4 model, as determined by the ModelFinder option within IQ‐TREE 2 [28]. From the initial dataset of 1316 genomes, two subsets were selected for Bayesian analysis: the first subset (*n* = 71) included our novel isolates belonging to the A2 lineage alongside reference strains, while the second subset (*n* = 14) consisted of lineage B3 genomes, also incorporating our novel isolates. Before temporal analysis, the strength of the molecular clock signal was assessed using the root‐to‐tip regression method available in TempEst v1.5.3 [29]. A time‐scaled phylogenetic tree was generated using BEAST v1.10.4, applying a relaxed lognormal clock model, a skyline population size prior, and the GTR substitution model [30]. The analysis used an empirical distribution of 1000 trees, running the MCMC chain for 20 million iterations, sampling every 2000. Maximum clade credibility (MCC) trees were summarized using TreeAnnotator v1.10.4 and visualized with the ggtree package in R [31].

### 2.5 Statistical Analysis

Chi‐square test or Mann–Whitney *U* test were employed to compare clinical features between EV/RV co‐infected and non‐co‐infected patients. Differences were considered significant at *p* < 0.05. Statistical tests were performed using GraphPad Prism 8 Software (GraphPad).

## 3 Results

### 3.1 Circulation of EV/RV

**Figure 1:**
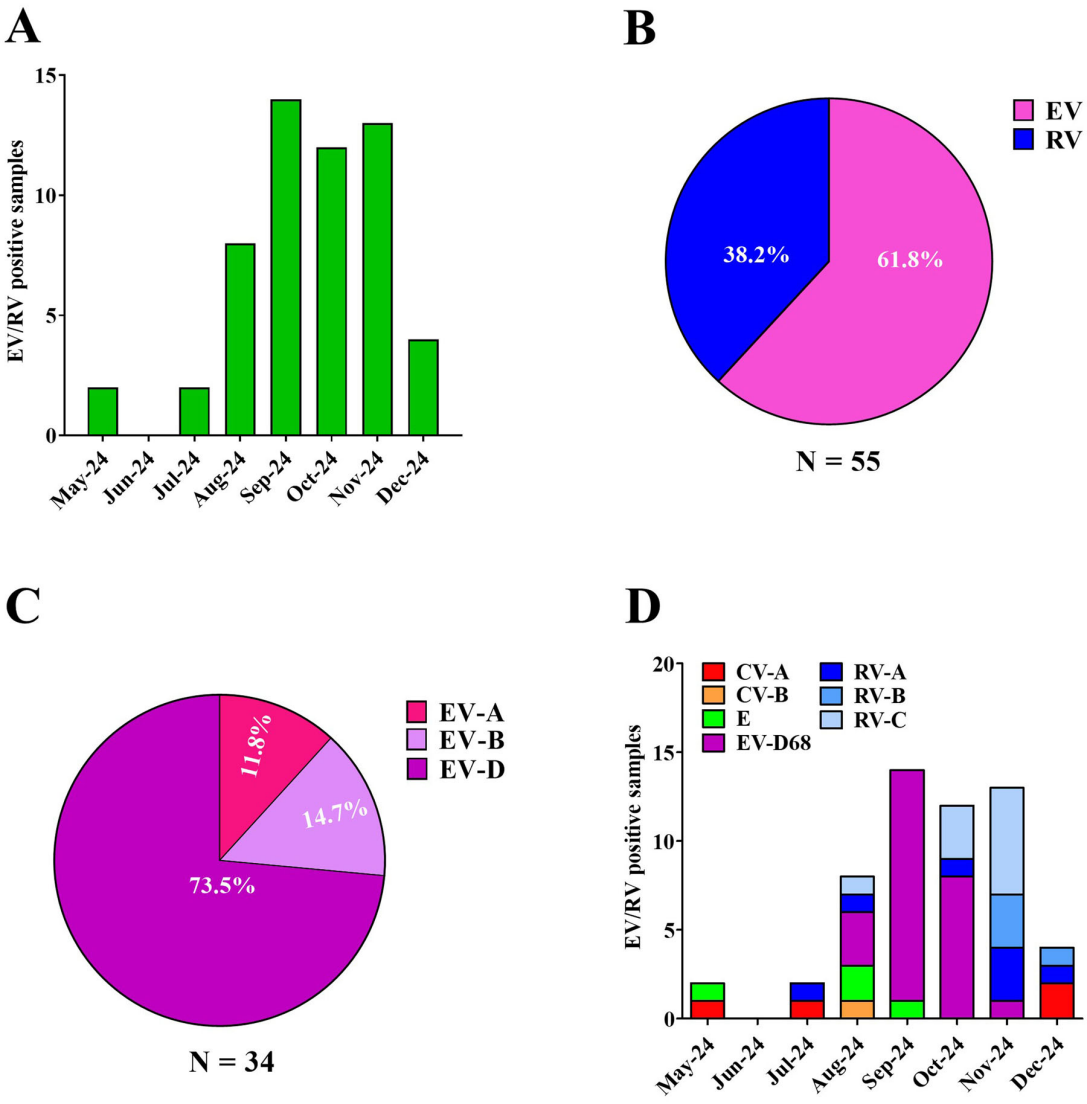
Distribution and frequency of EV/RV from May to December 2024 in the Brescia area. (A) The graph shows the spread of EV/RV‐positive cases in the pediatric population. (B) Pie chart displays the frequency of EV species as compared to RV ones. (C) Pie chart illustrates the percentage of each EV species. (D) The graph shows the monthly distribution, from May to December 2024, of EV/RV genotypes. RV genotypes are grouped according to their corresponding species (A–C). CV, coxsackievirus; E, echovirus; EV, enterovirus; RV, rhinovirus.

During the study period, a comprehensive analysis was conducted to evaluate the circulation of EV/RV. Specifically, along our surveillance program, we found 55 samples, collected from patients admitted at the ER or hospitalized at Brescia Civic Hospital, positive for EV/RV. As shown in Figure 1A, EV/RVs circulated at low levels from May to July 2024, while their incidence increased starting from August 2024, showing a peak in September, followed by a decline in December. These data suggest that symptomatic EV/RV infections occur in the pediatric population, with a higher incidence from late summer to fall.

To gain deeper insights into the circulation of EV/RV species in the Brescia area, each of the 55 EV/RV‐positive samples underwent NGS sequencing, followed by genotyping. Notably, we observed that 34 (61.8%) of the collected specimens belonged to EV species, while 21 (38.2%) were RV (Figure 1B). As shown in Figure 1C, among the EVs, EV‐D was the most prevalent (*n* = 25; 73.5%), followed by EV‐B (*n* = 5; 14.7%), and EV‐A (*n* = 4; 11.8%).

### 3.2 EV/RV Genotyping Revealed an EV‐D68 Outbreak

Subsequently, we further characterized EV/RV circulating in our geographical area, focusing on the different genotypes. We observed multiple EV genotypes co‐circulating in the Brescia area during the period under consideration. In particular, as shown in Figure 1D, we identified coxsackievirus A (CV‐A) among EV‐A species, coxsackievirus B (CV‐B) and echovirus (E‐9, E‐14, E‐18, E‐21, E‐25) among the EV‐B species.

Of interest, EV‐D68 was the only genotype found in EV‐D species. In particular, EV‐D68 genotype was first detected in the Brescia area in August 2024, accounting for 37.5% of the identified EV strains (Figure 1D). From this time until October, EV‐D68 incidence increased, reaching a peak in September and October (92.9% and 66.7%, respectively), becoming the predominant circulating EV genotype. As expected, in November 2024 a decrease in EV‐D68 incidence was observed (7.7%), while no EV‐D68 was detected in December. On the contrary, as shown in Figure 1D, the incidence of RV‐positive (RV‐A, RV‐B, and RV‐C) samples increased, reaching a peak in November and December (92.3% and 50.0%, respectively).

### 3.3 Epidemiological Analysis

**Figure 2:**
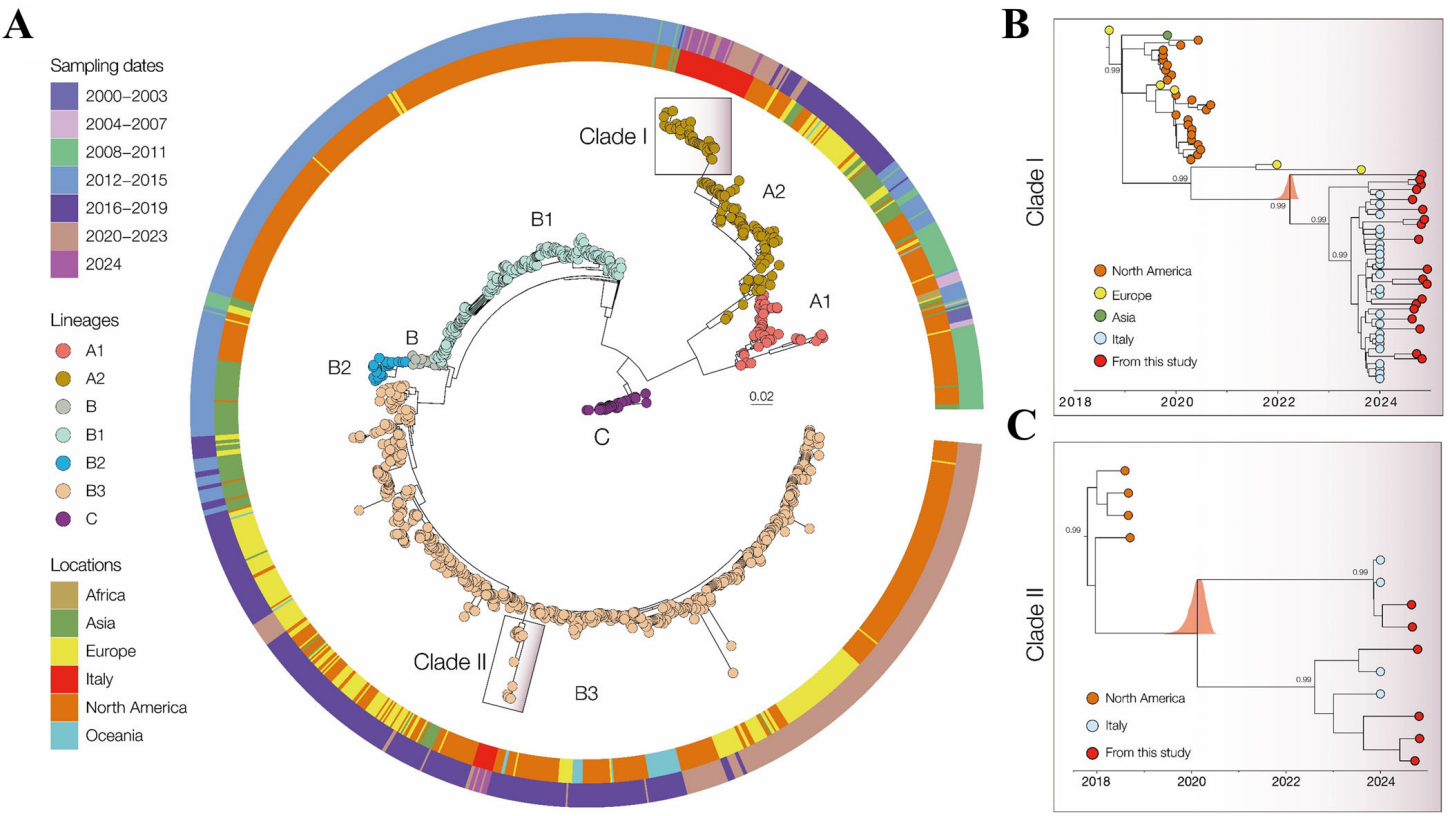
Phylogenetic relationships and temporal dynamics of EV‐D68. (A) Maximum likelihood (ML) phylogenetic tree of EV‐D68 genomes (*n* = 1316), including 25 novel Brescia isolates. Lineages are color‐coded. The heatmap surrounding the tree represents the geographic origin of the samples: Africa (brown), Asia (green), Europe (yellow), Italy (red), North America (gray), and Oceania (light blue). The outer ring indicates the sampling dates, ranging from 2000 to 2024. Two well‐supported monophyletic clades were identified: Clade I (A2) and Clade II (B3). (B) Bayesian MCC tree for Clade I (A2 lineage), showing Brescia isolates clustering with recent sequences from Europe, Asia, and North America. Posterior support values are indicated at key nodes. (C) Maximum clade credibility (MCC) tree for Clade II (B3 lineage), displaying a well‐supported cluster of Brescia isolates closely related to North American sequences. The shaded density plot represents the posterior distribution of the most recent common ancestor.

To properly define the evolutionary relationships on a global scale, a maximum likelihood (ML) phylogenetic tree was implemented. As shown in Figure 2A, the Italian samples evaluated in this study formed two well‐supported monophyletic clades (bootstrap = 1.0), corresponding to the A2 and B3 lineages. These findings align with recent reports [18], describing the diversification of EV‐D68 into distinct phylogenetic lineages. Moreover, the phylogenetic analysis highlights the global distribution of EV‐D68, with genomes originating from Africa, Asia, Europe, Italy, North America, and Oceania. The A2 lineage (Clade I, Figure 2B) predominantly comprises sequences from Europe, North America, and Asia, with many of the Brescia isolates from this study (*n* = 19; 26.8% of the analysed EV‐D68‐positive cases) clustering within the most recent temporal range (2024), indicating their introduction and ongoing circulation in the Country. The B3 lineage (Clade II, Figure 2C) includes genomes from North America and Italy, with the Brescia strains (*n* = 6; 42.9% of the analysed EV‐D68‐positive cases) forming a distinct, well‐supported cluster.

**Figure 3:**
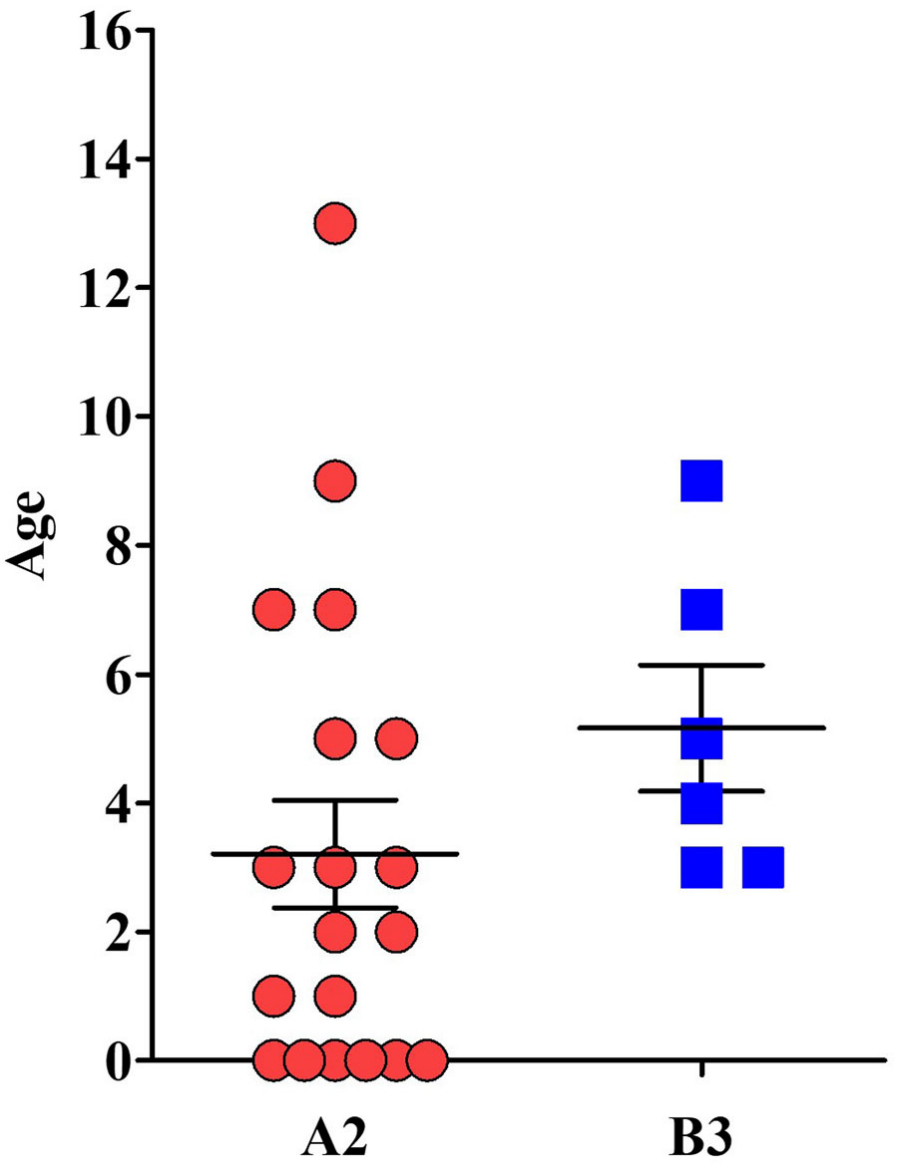
Age distribution of EV‐D68 A2 and B3 lineages. The graph represents the distribution of EV‐D68‐positive samples according to patients’ age for each lineage.

Sampling time distribution suggests that these lineages have been circulating since at least the early 2000s, with a notable increase in genomic diversity observed in recent years. To investigate the evolutionary dynamics of the two clades in greater detail, two subsets were analysed: 71 genomes for lineage A2 and 14 genomes for lineage B3. A strong correlation was observed between sampling date and root‐to‐tip genetic divergence in these datasets (Clade I: *r* ^2^ = 0.91, correlation coefficient = 0.96; Clade II: *r* ^2^ = 0.50, correlation coefficient = 0.70), indicating a relatively clock‐like evolutionary pattern. MCC tree (Figure 3B) estimated the mean time of origin for Clade I in Italy as June 2023 (95.0% highest posterior density [HPD]: August 2022 to September 2023). Within this clade, the novel Brescia isolates from 2024 clustered closely with contemporaneous strains, while basal sequences were represented by isolates from the Netherlands (2021) and Sweden (2023). For clade II (Figure 3C), the estimated mean time of origin was December 2022 (95% HPD: September 2018 to August 2023). In this clade, the novel Brescia isolates clustered with other recently detected Italian strains, whereas the basal sequences were represented by strains isolated in the USA in 2018. Taken together, these findings suggest a recent introduction of both lineages in Italy, with ongoing diversification and continued viral circulation.

EV‐D68 lineage A2 was predominantly sampled in 19/25 children (76.0%, median age 3 years), while EV‐D68 lineage B3 in 6/25 children (24.0%, median age 5 years) (Figure 3). According to the EV‐D68 epidemic course, both lineages were detected at the highest frequencies in September 2024, with no statistically significant difference in their monthly distribution.

### 3.4 Clinical Features of EV‐D68‐Positive Children

On the whole, 25 out of 55 (45.5%) pediatric cases were EV‐D68‐positive, with a mean age of 4 years. As documented in Table 1, clinical manifestations in this population spanned from mild to severe symptoms. In fact, only 12 out of 25 (48.0%) children were hospitalized with an average hospital stay of 4 days. Among these, the pediatric intensive care unit (PICU) was necessary for 3 out of 25 (12%) with one child showing clinical signs of meningitis (ID10), while the others were affected by acute respiratory insufficiency. Specifically, ID106 presented pneumomediastinum and ID102, an ex‐premature boy, experienced convulsive *epilepticus status*.

**Table 1:** Clinical features of pediatric EV‐D68‐positive patients.

Patient	EV‐D68 lineage	Age	Symptoms	Comorbidities	Co‐infections	Hospital admission	PICU admission	Hospital stay (day)	Laboratory abnormalities
ID99	A2	6 years	Acute bronchitis, wheezing	—	*Moraxella catarrhalis*	Yes	No	1	l‐ymphopenia (880 cells/µL)
ID102	B3	5 years	Acute respiratory insufficiency, convulsive epilepticus status	Encephalopathy	*Haemophilus influenzae*	Yes	Yes	1	—
ID18	A2	2 months	Acute upper airways infection	Gastroesophageal reflux	—	Yes	No	1	neutropenia (550 cells/µL)
ID103	B3	3 years	Acute bronchitis, wheezing	—	—	Yes	No	—	—
ID107	A2	4 years	Acute bronchitis, wheezing	Allergic asthma	—	Yes	No	7	lymphopenia (650 cells/µL)
ID42	A2	3 years	Pneumonia	Congenital encephalopathy	—	Yes	No	6	—
ID43	A2	1 year	Acute bronchitis, wheezing	—	—	No	No	—	—
ID44	A2	8 years	Acute upper airways infection	—	—	No	No	—	—
ID45	A2	4 years	Acute bronchitis, wheezing	Allergic asthma	—	No	No	—	—
ID47	A2	6 months	Acute upper airways infection, otitis media	Alloimmune neutropenia	—	Yes	No	3	—
ID48	A2	2 years	Acute bronchitis, wheezing	—	RV‐C, SARS‐CoV‐2	No	No	—	—
ID49	A2	1 month	Acute upper airways infection	—	RV‐A, RV‐C	No	No	—	—
ID01	A2	5 months	Acute upper airways infection	—	—	Yes	No	3	—
ID02	A2	8 years	Pneumonia	Congenital encephalopathy	—	No	No	—	—
ID03	A2	6 months	Acute upper airways infection	—	RV‐C	No	No	—	—
ID04	A2	3 years	Acute bronchitis, wheezing	Ex‐preterm (32 weeks)	RV‐A	No	No	—	—
ID106	B3	8 years	Pneumomediastinum	Allergic asthma	RV‐A	Yes	Yes	9	—
ID119	A2	3 years	Acute bronchitis, wheezing	Atopy	RV‐C, *Haemophilus influenzae*	Yes	No	4	—
ID140	B3	6 years	Acute bronchitis, wheezing	—	*Mycoplasma pneumoniae*	Yes	No	6	lymphopenia (860 cells/µL)
ID05	A2	5 months	Bronchiolitis	—	—	No	No	—	—
ID06	B3	3 years	Acute bronchitis, wheezing	—	RV‐C	No	No	—	—
ID07	A2	5 months	Bronchiolitis	—	RV‐B	No	No	—	—
ID08	B3	3 years	Acute bronchitis, wheezing	—	—	No	No	—	—
ID09	A2	12 years	Acute bronchitis, wheezing	Atopy	RV‐C	Yes	No	3	neutropenia (660 cells/µL)
ID10	A2	6 years	Meningitis	—	RV‐C, E9	Yes	Yes	3	—

Abbreviations: E, echovirus; PICU, pediatric intensive care unit; RV, rhinovirus.

The most common clinical sign was acute wheezing, observed in 12 out of 25 patients (48.0%), including children with predisposing co‐morbidities: 5 children (ID107, ID45, ID106, ID119, ID09) with a known history of allergic asthma or atopy (age range: 3–13 years) and a 3‐year‐old boy (ID04), who was born at 32 weeks of gestation, had neonatal respiratory distress and suffered from recurrent airways infections. Furthermore, EV‐D68 infection presented with bronchiolitis in 2 babies (ID05 and ID07), with acute upper airways infection in other 6 patients (including ID47, a one 6‐month‐old boy who developed acute otitis media as a complication), or with pneumonia in 2 subjects (ID02 and ID42), who had congenital encephalopathy.

Blood test was performed in 14 out of 25 (56.0%) patients, revealing a total white blood count of 12.40 ± 3.22 cells/µL (mean ± SD), an absolute neutrophil count (ANC) of 7.54 ± 4.78 cells/µL and an absolute lymphocyte count (ALC) of 2.86 ± 1.52 cells/µL. Neutropenia was observed in only 2 patients (ID09 and ID18) (ANC range: 560–660 cells/µL), and lymphopenia was detected in just 3 patients (ID99, ID107, ID140) (ALC range: 650–880 cell/µL), suggesting that these two conditions were not related to a more severe outcome. C‐reactive protein, tested in 16 out of 25 (64.0%) patients, showed mild to moderate values (mean ± SD 20 ± 25 mg/L). Bacterial culture of the NPA from 4 out of 25 (16.0%) EV‐D68‐positive pediatric patients revealed bacterial co‐infections, including *Haemophilus influenzae* (ID102 and ID119), *Moraxella catarrhalis* (ID99) and *Mycoplasma pneumoniae* (ID140). Oxygen therapy, during hospital stay, was required for 7 out of 25 (28.0%) patients. All the individuals received supportive treatments, including salbutamol inhalers, antipyretic medications and antibiotics, when a bacterial infection was diagnosed.

### 3.5 EV‐D68 Co‐Infections

The majority of the analyzed samples did not present any co‐infection (15/25, 60.0%, Table 1). Interestingly, in 10 out of 25 (40.0%) pediatric cases, EV‐D68 was detected with at least one other EV/RV. Among the 10 co‐infections, the 80.0% was characterized by the presence of two genotypes, while the 20.0% by three genotypes. In detail, RV‐C was the most frequently co‐infecting virus (60.0%), followed by RV‐A (30.0%), and RV‐B or E (10.0%). Although influenza viruses and SARS‐CoV‐2 are known to circulate during November and December 2024, we reported only one co‐infection with SARS‐CoV‐2, and specifically in a EV‐D68‐positive child (ID48) also co‐infected with RV‐C.

Concerning the EV‐D68 outbreak, 50.0% of co‐infections were detected in October 2024, 40.0% in September, and 10.0% in November. Except for the 3‐year‐old baby (ID10), who developed meningitis, no relevant differences in clinical manifestation between co‐infected and non‐co‐infected patients were reported (Table 2). Focusing on the ID10 case, NGS analysis identified three different EVs in the CSF: EV‐D68, RV‐C and echovirus E‐9. Since EV‐D68 and echovirus E‐9 are both associated with respiratory and neurological manifestations [32], we cannot exclude their mutual contribution to clinical symptoms and signs.

**Table 2:** Clinical features of EV‐D68‐infected and EV/RV‐co‐infected pediatric patients.

Pediatric EV‐D68 patients	EV‐D68	EV‐D68 co‐infected	*p* value
Mean age	3	4	0.88
Clinical manifestations			
Wheezing	7	5	0.07
Acute bronchitis	7	5	0.07
Acute respiratory insufficiency	1	0	
Convulsive epilepticus status	1	0	
Acute upper airways infection	4	2	0.12
Pneumonia	2	0	
Otitis media	1	0	
Pneumomediastinum	0	1	
Bronchiolitis	1	1	0.76
Meningitis	0	1	
PICU admission	1	2	0.80
Length of hospital stay (median days)	3.6	4.7	0.51

## 4 Discussion

During COVID‐19 pandemic, EV/RV were found to be the respiratory viruses that predominantly co‐circulated with SARS‐CoV‐2 [33, 34]. Following the withdrawal of preventive measures adopted against SARS‐CoV‐2 the incidence of EV/RV remained consistently higher as compared to the pre‐pandemic era. These data reveal a shift in the circulation patterns of common respiratory viruses, contributing to the emergence and re‐emergence of uncommon ones.

In this study based on the retrospective analysis of EV/RV positive samples collected from pediatric patients visiting the ER or hospitalized at a single center in Brescia, we highlighted an EV‐D68 outbreak occurred from August to November 2024. The rapid upsurge of EV‐D68‐positive cases in Lombardy in August and September 2024 has already been reported by Pariani and colleagues [18]. However, our data described the whole course of the EV‐D68 epidemic from the rapid rise to the decline of EV‐D68‐positive cases, focusing on the clinical manifestations of EV‐D68‐positive children. As typical in temperate regions [1], EV‐D68 was first detected in late summer, reaching a peak in September–October 2024 and then declining in November. In our cohort, as typical of EV‐D68 respiratory infections, symptoms spanned from mild to severe [1], where asthma represented the most common comorbidity which could contribute to the development of wheezing [35, 36]. The median age of EV‐D68 infection in our study was 4 years, which agrees with the average age reported in most previous studies (3–5 years) [3, 19, 20, 21, 37]. This pattern aligns with previously reported outbreaks, showing a concentration of EV‐D68 cases in summer and early autumn, thus supporting the evidence that the epidemiological characteristics of the Brescia outbreak in 2024 is not dissimilar from the previous ones in the literature.

In accordance with previous studies conducted in Lombardy [17, 18], in our study none of the EV‐D68 patients showed signs or symptoms of AFM or AFP. However, we reported an episode of meningitis in a 9‐year‐old boy without any comorbidity but co‐infected by EV‐D68, RV‐C and echovirus E‐9. In this context, determining the virus responsible for the clinical symptoms and, especially, for neurological manifestations is particularly challenging. Indeed, both EV‐D68 and echovirus E‐9 have been previously associated with episodes of meningitis and encephalitis, a finding that complicates interpretation of the clinical picture [32, 38, 39, 40, 41]. Since the neurological involvement in the EV‐D68‐positive patients pertained to this isolated case, we can speculate that, despite changes in clades and subclades of circulating viruses, the clinical features remained similar to those reported in 2024 in Europe, in contrast to those recorded in 2014 and 2016 in the United States and Canada.

Interestingly, in this study we highlight the presence of viral co‐infections, mostly RVs, in 40% of the EV‐D68‐positive pediatric patients. This evidence goes hand in hand with the knowledge that in temperate regions, although circulating at low levels throughout all the year, EVs and RVs spread in the population following a seasonal pattern: EV infections occur at high levels in late summer and fall, while RVs in spring and fall [42, 43, 44]. Hence, during autumn, especially when their incidence is elevated, EV and RV circulation could overlap, leading to co‐infection in the same individual. Accordingly, 50% of co‐infections were detected in October 2024, when the EV‐D68 outbreak peaked and the rise of RV infections was observed. In particular, we found that the co‐infecting virus in 90% of cases belonged to RV species (RV‐A, RV‐B, or RV‐C), while in 10% to E‐9. Interestingly, comparative analysis revealed no statistical difference in clinical manifestations between patients with EV‐D68 mono‐infection and those with viral co‐infection. This finding points to a key role of EV‐D68 in mild‐to‐severe respiratory infections. However, its role in co‐infections in the absence of a quantitative result assessing its predominancy in the biological sample, cannot be established. A thorough clinical analysis of EV‐D68‐infected patients in the presence of EV/RV co‐infections is needed to better understand the real involvement of EV‐D68 in the clinical manifestation and how other viral pathogens may interact with it and become synergic, competitive or silent partners during viral infection.

Additionally, 16% of EV‐D68‐positive pediatric acute severe respiratory infections, were characterized by co‐infections with bacterial respiratory pathogens. In this regard, further investigations on EV‐D68 and bacterial co‐infections are necessary to shed light on the contribution to clinical symptoms and host immune response of each pathogen.

Differently to the latest European epidemic dominated by EV‐D68 B3 strains, but in agreement with Pariani et al. [18], our phylogenetic analysis revealed that EV‐D68 lineage A2 represented the most prevalent circulating strain during the 2024 outbreak. However, as already reported by Poelman and colleagues in 2015 [35], no significant differences in clinical manifestations, hospitalization, bacterial or viral co‐infections, were found between EV‐D68 A2 and B3 lineages.

Nonetheless, our study presents at least four limitations. First, the qualitative nature of the real time PCR employed to detect EV‐D68‐positive cases does not provide information about viral load, which could contribute to the understanding of the clinical picture. Second, the targeted NGS approach focus on the VP2‐VP4 region, due to the extreme variability of EV genomes, could not be sufficient to accurately assess the exact contribution of EV‐D68 in EV/RV co‐infections. Furthermore, in patients presenting mild respiratory infections in the presence of bacteria, it is more likely that the latter are responsible for the clinical symptoms. However, since we can only speculate on this aspect, further studies employing a more sensitive approach are necessary to clarify the contribution of each pathogen and their mechanisms of interaction in infected individuals. Third, we focused on a specific set of samples obtained from patients visiting the ER or hospitalized, collected from a single center in Brescia from May to December 2024, disregarding EV‐D68 circulation outside. Therefore, a more comprehensive analysis, involving different backgrounds, would be critical to define the extent of EV‐D68 spread. Fourth, we included only patients with clinical symptoms, thus the overall pathogenicity of EV‐D68 is likely to be overestimated.

On the whole, our study describes the EV‐D68 outbreak which took place in Brescia from August to November 2024. Furthermore, although we did not detect any case showing signs of neurological impairment, our study emphasizes the crucial importance of the laboratory‐based surveillance program of EV/RV respiratory infections, followed by genomic surveillance, to monitor the circulating strains and their evolution, and to understand their impact on the public health system and on the community. These insights will help the prompt adoption of preventive measures to counteract viral spread, the rapid detection of possible new emerging strains, and also the protection of the most vulnerable individuals.

## Author Contributions

A.C. and F.C. initiated the idea. A.C., F.C., M.C., and R.B. wrote and reviewed the manuscript. S.M., A.B., and L.D. wrote the original draft. S.M., A.B., and L.D. performed data acquisition. S.M., A.B. L.S., and G.V performed laboratory analysis. M.G. performed phylogenetic analysis. S.M. and A.B. performed statistical analysis. L.D. and R.B. were involved in patient care. All authors contributed to all sections relevant to their experience and helped finalize the text and content.

## Ethics Statement

The study was conducted in strict adherence to the Helsinki Declaration. Local ethical committee approval PNCPED2019—NP 3460.

## Conflicts of Interest

The authors declare no conflicts of interest.

## Data Availability

The data that support the findings of this study are available from the corresponding author upon reasonable request. Sequences obtained in this study are available on request.
